# An improved ternary vector system for *Agrobacterium*-mediated rapid maize transformation

**DOI:** 10.1007/s11103-018-0732-y

**Published:** 2018-04-23

**Authors:** Ajith Anand, Steven H. Bass, Emily Wu, Ning Wang, Kevin E. McBride, Narayana Annaluru, Michael Miller, Mo Hua, Todd J. Jones

**Affiliations:** 1 Corteva Agriscience™, Agriculture Division of DowDuPont™, 8305 NW 62nd Avenue, Johnston, IA 50131 USA; 2 Corteva Agriscience™, Agriculture Division of DowDuPont™, 4010 Point Eden Way, Hayward, CA 94545 USA; 3 Present Address: 1969 West Grand Canyon Drive, Chandler, AZ 85248 USA

**Keywords:** *Agrobacterium*, Accessory plasmids, Ternary vector, Rapid maize transformation, Origins of replication, pVIR plasmids, Babyboom, Wuschel

## Abstract

**Key message:**

A simple and versatile ternary vector system that utilizes improved accessory plasmids for rapid maize transformation is described. This system facilitates high-throughput vector construction and plant transformation.

**Abstract:**

The super binary plasmid pSB1 is a mainstay of maize transformation. However, the large size of the base vector makes it challenging to clone, the process of co-integration is cumbersome and inefficient, and some *Agrobacterium* strains are known to give rise to spontaneous mutants resistant to tetracycline. These limitations present substantial barriers to high throughput vector construction. Here we describe a smaller, simpler and versatile ternary vector system for maize transformation that utilizes improved accessory plasmids requiring no co-integration step. In addition, the newly described accessory plasmids have restored virulence genes found to be defective in pSB1, as well as added virulence genes. Testing of different configurations of the accessory plasmids in combination with T-DNA binary vector as ternary vectors nearly doubles both the raw transformation frequency and the number of transformation events of usable quality in difficult-to-transform maize inbreds. The newly described ternary vectors enabled the development of a rapid maize transformation method for elite inbreds. This vector system facilitated screening different origins of replication on the accessory plasmid and T-DNA vector, and four combinations were identified that have high (86–103%) raw transformation frequency in an elite maize inbred.

**Electronic supplementary material:**

The online version of this article (10.1007/s11103-018-0732-y) contains supplementary material, which is available to authorized users.

## Introduction

*Agrobacterium*-mediated plant transformation is a preferred method for plant genetic engineering since it was known to reliably transfer large (30–150 kb) DNA fragments into plants to generate events with low transgene copy number (Gelvin 2003, 2009; Komari et al. 2004). Early attempts to exploit *Agrobacterium* as a natural tool for transgenesis required introducing a target gene directly into the transfer DNA (T-DNA) region of the large tumor-inducing (Ti) plasmid either through a single or double-homologous recombination. This was challenging given the large size of the Ti plasmid (Fraley et al. 1985, 1983; Zambryski et al. 1983). The complexity of plasmid construction was later solved through the development of the ‘binary vector’ system, where the T-DNA is separated on a smaller, independent episome from the virulence genes encoded by a disarmed Ti plasmid (Bevan 1984; Hoekema et al. 1983). This binary system is routinely used for plant genetic engineering.

Gene dosage effects have been observed with some of the virulence genes (Jin et al. 1987) which led to the development of a superbinary vector, pSB1. Plasmid pSB1 carries a DNA fragment (~ 14.8 kb) with *vir* genes (B, G, part of C and D) from the Ti plasmid, pTiBo542 (Komari 1990). Application of the pSB1 superbinary system broadened the host range of plants amenable to transformation with *A. tumefaciens* (Cheng et al. 1997; Hiei et al. 1994; Ishida et al. 1996; Komari et al. 1996, 2004, 2006; Tingay et al. 1997) and is widely regarded as the primary choice of vector system for maize transformation (Cho et al. 2014; Zhi et al. 2015). However, the large size of the plasmid pSB1 (~ 37 kb) and the co-integration step required for introduction of the T-DNA vector complicates vector construction and structural confirmation of the plasmid. Additionally, pSB1 contains a tetracycline resistance gene (*tetAR*) for selection and C58-based *Agrobacterium* strains are known to give rise to spontaneous tetracycline resistant mutants (Luo and Farrand 1999). The plasmid pSB1 was also found to have a defective set of virulence genes (specifically a frame-shift in *virC1* gene and a truncated *virD* operon), repetitive and non-essential DNA sequences, and a large fragment containing the RK2 origin of replication (ORI), which we speculate negatively impacted plasmid function and plant transformation.

In this report, we describe the development of an improved ternary vector system for crop transformation using the newly described accessory “pVIR” plasmids. The pVIR plasmids have many desirable features, such as small size, enhanced vector stability, an improved bacterial selectable marker, and amended *vir* genes (operons *virC, virD* and *virE*) for improved T-DNA delivery (Anand et al. 2017). Introduction of these accessory plasmids *in trans* with the T-DNA binary vectors in the same *Agrobacterium* strain facilitated the generation of highly versatile ternary vectors (Supplementary Fig. 1). Ternary vectors harboring a plasmid containing a constitutive *virG* mutant (*virG*N54D) have been described that efficiently transfer T-DNA in multiple dicot plant species (Kikuchi et al. 2005; Fits et al. 2000). The introduction of pVIR plasmids in the ternary vector design nearly doubled raw transformation rates and improved the number of transformation events of usable quality in difficult-to-transform maize genotypes. Incorporation of the ternary vector design enabled the development of a rapid maize transformation system (Lowe et al. 2018) that relies on expression of morphogenic genes *Baby boom* (*Bbm*) and *Wuschel* (*Wus2*) (Lowe et al. 2016), for rapid recovery of stable transgenic plants. We describe testing of different ORI in the accessory plasmid and T-DNA plasmid using the rapid transformation system. Through the testing, we identified unique ORI-by-ORI combinations with relatively high raw transformation frequencies: the percent of infected embryos producing stable events was 86–103%. This represents a six-to-seven-fold improvement over conventional random transformation (13–14%) using the plasmid pSB1 in an elite maize inbred.

## Results and discussion

### Annotation of pSB1

**Fig. 1:**
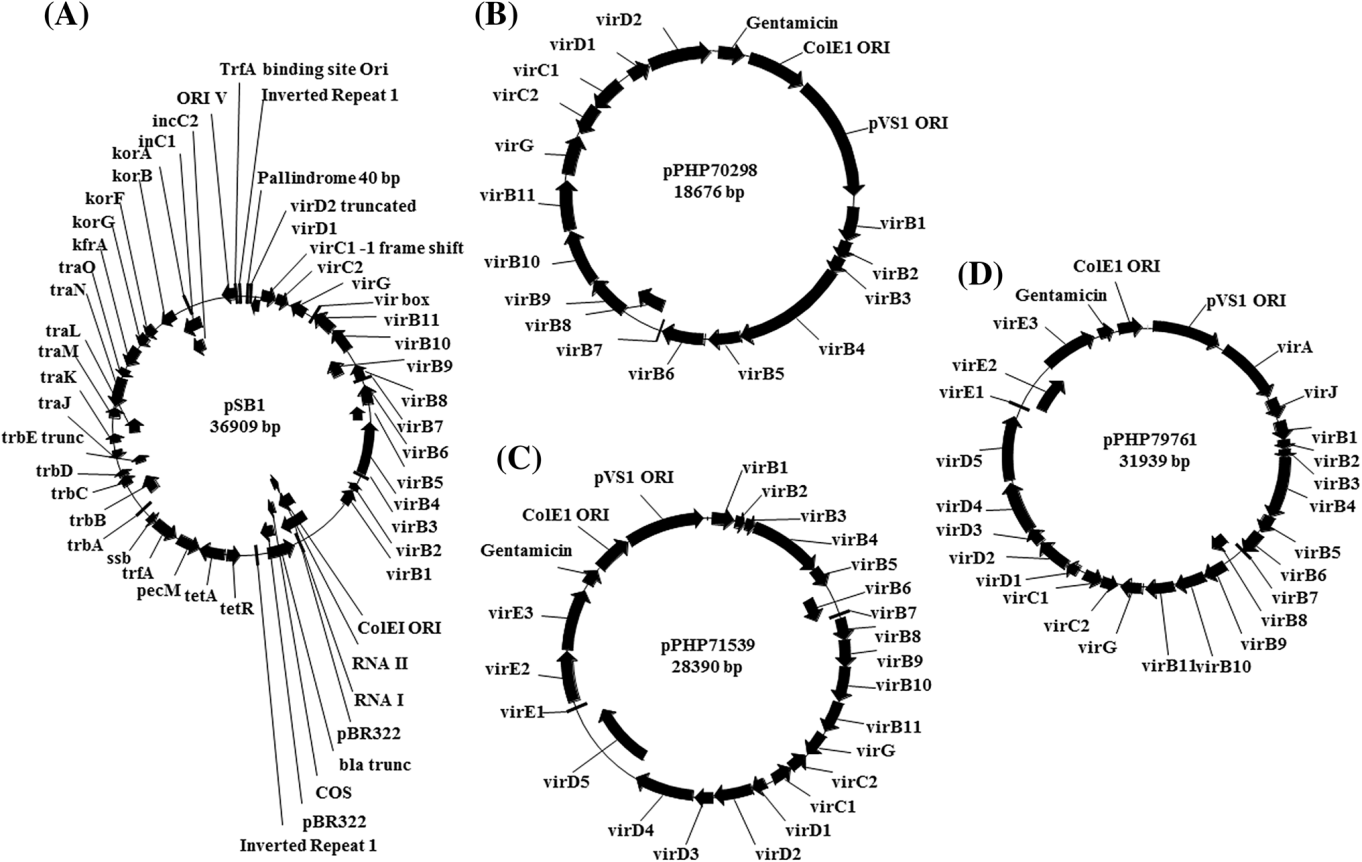
The maps of accessory plasmids described in this study, **a** complete annotation of the pSB1 vectors (Komari et al. 1996) following de novo assembly of the sequenced plasmid, **b**–**d** newly described pVIR plasmids, pPHP70298, pPHP71539 and pPHP79761 respectively, with complete annotation

We generated a complete annotated map of the pSB1 vector (Fig. 1a) using the published sequence (GenBank: AB027255.2) and sequence assembled de novo. As shown in Fig. 1a, plasmid pSB1 is ~ 37 kb and includes a 14.8 kb KpnI fragment with *vir* genes from Ti plasmid pTiBo542 that enable transformation of a broad range of crops (Cheng et al. 1997; Hiei et al. 1994; Ishida et al. 1996; Tingay et al. 1997). pSB1 was found to have a 19.7 kb fragment containing the ORI and partitioning functions from the broad host range plasmid RK2 for replication in *Agrobacterium* (Knauf and Nester 1982; Schmidhauser and Helinski 1985). The assembled sequence identified several nonessential elements including a ~ 14 kb sequence between the trfA replication protein from its origin of replication target site that included some *tra* and *trb* genes encoding a non-functional conjugative transfer system. A 2.7 kb pBR322 fragment containing the ColE1 ORI and an inactive *β*-*lactamase* coding sequence with the embedded lambda phage COS site was also identified. Additionally, the ColE1 ORI (1.0 kb) was introduced with a 2.7 kb fragment from plasmid pBR322 with non-essential sequences. We also identified a 146 bp inverted repeat and a 32 bp palindrome of unknown origin in the vector backbone (Fig. 1a).

### Overview of the pVIR accessory plasmids

A series of small and versatile accessory plasmids (Fig. 1b–d) (Anand et al. 2017) were designed with the intent to simplify and optimize *Agrobacterium*-mediated crop transformation. These plasmids, designated pVIR referred to as pPHP70298, pPHP71539 and pPHP7976, contain sets of different *vir* genes (*virG, virE, virA, virJ, virB, virC*, and *virD*) from the hypervirulent pTiBo542 plasmid, as well as a stable high copy number pVS1 ORI (Zhi et al. 2015) and gentamicin bacterial selection marker. The respective sequences can be found under GenBank accession numbers MF788072, MF788073 and MF788074. The accessory plasmids were generated to support a ternary vector design for crop transformation (Supplementary Fig. 1) and to simplify vector assembly.

Design of the pVIR plasmids was based on the open source superbinary vector pCAMBIA5105 (http://www.cambia.org/daisy/cambia/585). The large ORI (19.7 kb) containing the moderate copy plasmid replicon RK2 ORI (10–12 copies) and partitioning sequences in pSB1 (Komari et al. 1996) was replaced with pVS1, a smaller (~ 2.6 kb), more stable (Stanisich et al. 1977) and high copy (~ 20 copies) ORI (Zhi et al. 2015). The choice of plasmid ORI is an important determinant of plasmid size, stability, copy number, host specificity and compatibility with other ORI when additional plasmids are present in the same bacterial cell. The original RK2 ORI is likely to have adversely affected plasmid performance due to its large size and the presence of intervening non-essential DNA sequence. In a previous study, we demonstrated the choice of ORI directly impacted maize transformation (Zhi et al. 2015).

We corrected several deficiencies in the virulence genes in plasmid pSB1. The single base pair frameshift mutation in the *virC1* gene was replaced with a functional *virC* operon consisting of the *virC1* and *virC2* genes. The original frame-shift in *virC1* likely resulted in an inactive VirC protein. The VirC protein is known to bind to the *cis-*element “over-drive” sequences for processing of single stranded T-DNA and translocation of the T-DNA to the poles for enhanced T-DNA delivery (Close et al. 1987; Lu et al. 2009). The truncated *virD2* gene in pSB1 was also replaced with a functional *virD2* gene as in pPHP70298 (Fig. 1b). The VirD1 and VirD2 endonuclease complex is essential for T-DNA strand synthesis, processing and transfer (reviewed in Gelvin 2003; Tzfira and Citovsky 2006). With the addition of a more stable ORI and fully functional *vir* genes, the plasmid pPHP70298 is an improved version of plasmid pSB1, but only half the size (~ 19 kb).

**Fig. 2:**
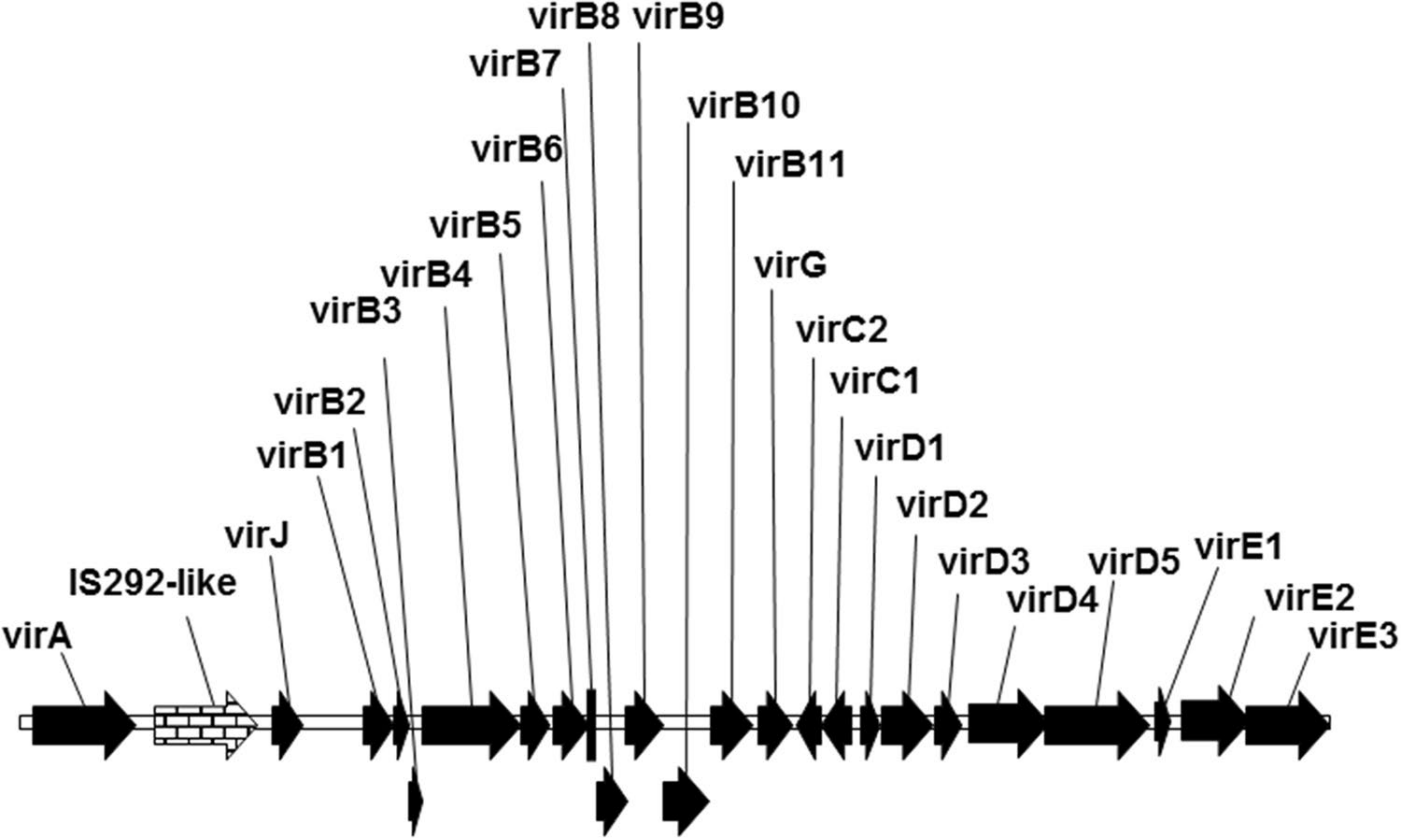
The source of *vir* genes and their arrangement on the Ti plasmid pTiBo542. A 3.2 kb fragment including the IS292-like insertion element between *virA* and *virJ* and an 8.1 kb fragment between *virJ* and *virB1* were deleted from the *vir* gene cluster in plasmid pPHP79761

Plasmid pPHP71539 (Fig. 1c) was designed to contain additional *vir* genes including the complete *virD* operon (i.e., *virD1-D5*) and *virE* operon (*virE1-3*), that are both lacking in pPHP70298. The *vir* gene composition in plasmids pPHP70298 and pPHP71539 (Fig. 1b, c) includes only the *vir* coding sequences (with intervening non-coding sequences omitted) from a 31.5 kb fragment present in pTiBo542 plasmid (GenBank Accession No. NC_010929.1, Fig. 2). However, plasmid pPHP79761 (Fig. 1d) contains additional essential Vir operons from pTiBo542 including *virA, virJ, virB, virG, virC, virD and virE*, associated with plant transformation (reviewed in (Nester 2014)). A 3.2 kb fragment including the IS292-like insertion element (Ponsonnet et al. 1995) between *virA* and *virJ* and an 8.1 kb fragment between *virJ* and *virB1* were deleted from the *vir* gene cluster in plasmid pPHP79761 (Fig. 2). Importantly, even with the added features, the newly described pVIR plasmids (pPHP70298, pPHP71539 and pPHP79761) are all smaller than pSB1 (Fig. 1).

Additional improvements incorporated in the pVIR plasmids included elimination of the 7.0 kb truncated *tra* and *trb* operons or flanking genes within the original 19.7 kb RK2 ORI, a 40 bp palindromic sequence and two copies of the 146 bp inverted repeat sequence on pSB1 (Fig. 1A). The 2.7 kb pBR322 fragment present in pSB1 that contains the ORI, an inactive *β*-*lactamase* coding sequence, and 18 bp poly-G flanked lambda COS sites were removed and replaced with the 1.2 kb ColE1 ORI. The tetracycline efflux transporter *tet*A gene, a potentially problematic marker in C58-based *Agrobacterium* strains (Luo and Farrand 1999), was replaced with a stringent selectable marker GmR (synthetic *aacC1*) conferring resistance to gentamicin. The plasmid containing this gentamicin selectable marker was highly stable in *E. coli* and multiple *Agrobacterium* strains AGL0, AGL1 and LBA4404; data from LBA4404 are presented below.

### pVIR plasmids are stable

**Fig. 3:**
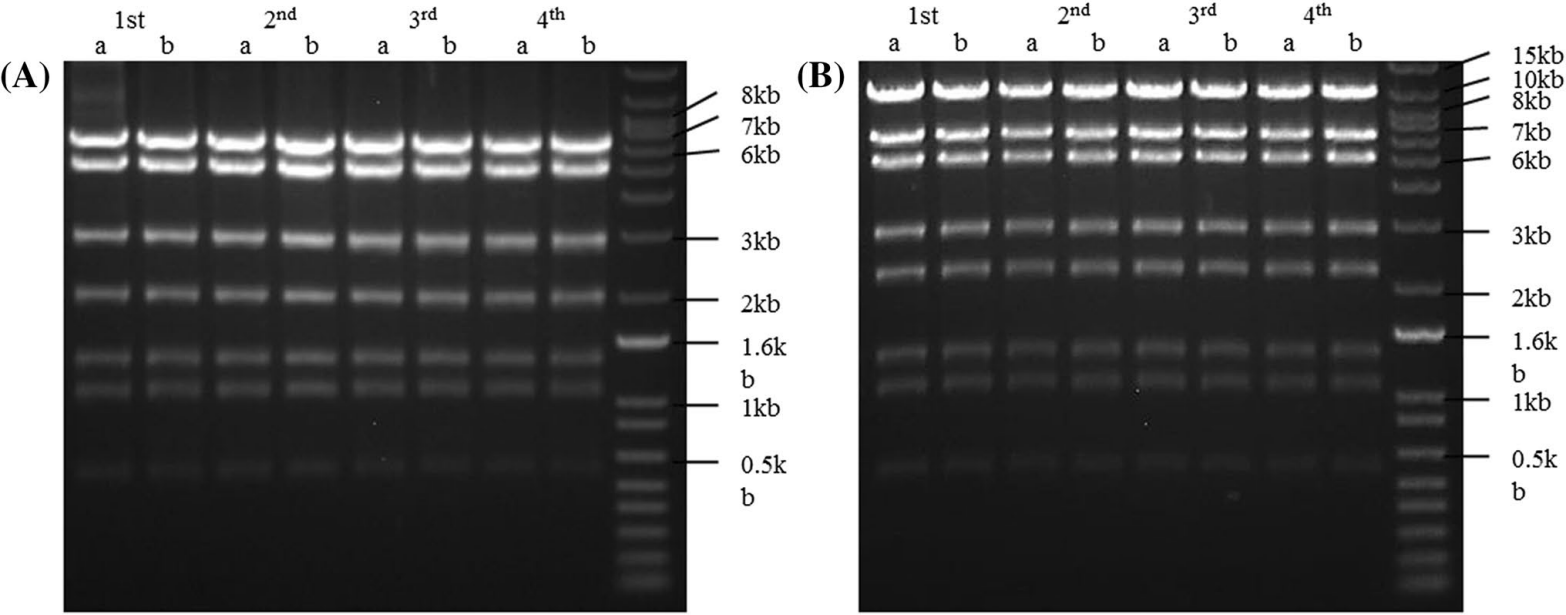
Restriction mapping of the plasmid DNA in *Agrobacterium tumefaciens* to determine plasmid integrity. *Agrobacterium* strain LBA4404THY- harboring the plasmid pPHP70298 (**a**) or pPHP71539 (**b**) was isolated after four consecutive passage (day 1–4), and retransformed into *E. coli*. Pooled plasmid DNA from two independent *E. coli* passages (*a* and *b* colonies used for transforming *Agrobacterium* cells, day 1–4) was subjected to restriction enzyme digestion to determine plasmid integrity. The DNA banding pattern for PstI showed no detectable differences between the two colonies for all the time points. The plasmid DNA was further sequence confirmed by next generation sequencing (Illumina)

The stable maintenance and integrity of plasmids pPHP70298 and pPHP71539 in the different bacterial backgrounds (*E. coli* and *Agrobacterium*) were determined. Two bacterial strains, *E. coli* and an auxotrophic (thymidine mutant, THY-) strain of *Agrobacterium* LBA4404THY- containing the pVIR plasmids, were grown as described in the Materials and Methods. The cultures after each passage (i.e., overnight culture) were serially diluted and plated on selective and non-selective media, followed by counting the numbers of colonies on each of the plates, respectively. A difference of less than 10% was observed in the colony numbers in the serial dilution plating, suggesting that both plasmids were highly stable in the two different bacterial strains. The integrity of the pVIR plasmids was inferred by restriction enzyme analysis of the pooled plasmid DNA extracted from two independent passages in LBA4404THY- and transformed into Top10 cells (Fig. 3). The DNA banding pattern across multiple enzyme digests (with PstI, BglII, NheI, BamHI and PvuII) was unchanged between the two independent *E. coli* passages (day 1–4) for both pPHP70928 and pPHP71539. A representative experiment showing the restriction digest fragments of pPHP70928 and pPHP71539 using PstI is presented in Fig. 3a, b. The same plasmid DNA was further analyzed by next-generation sequencing (Illumina), and the stability of the plasmids was confirmed at the base pair level by sequence alignment of the expected and observed bases (Supplementary Fig. 2).

### An improved ternary vector system for maize transformation

**Fig. 4:**
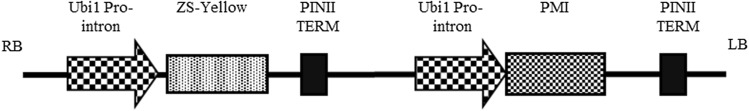
Schematic representation of the T-DNA elements on the binary plasmid pPHP45981 used in the ternary vector with the different accessory plasmids, pSB1 and pPHP70298 for transforming maize inbred lines HC69 and PH2RT. RB right border, Ubi1 Pro-intron maize ubiquitin 1 promoter-intron, ZS-yellow fluroscent protein (YFP), PMI phosphomannose isomerase, PINII TERM potato proteinase inhibitor II terminator, LB left border

**Fig. 5:**
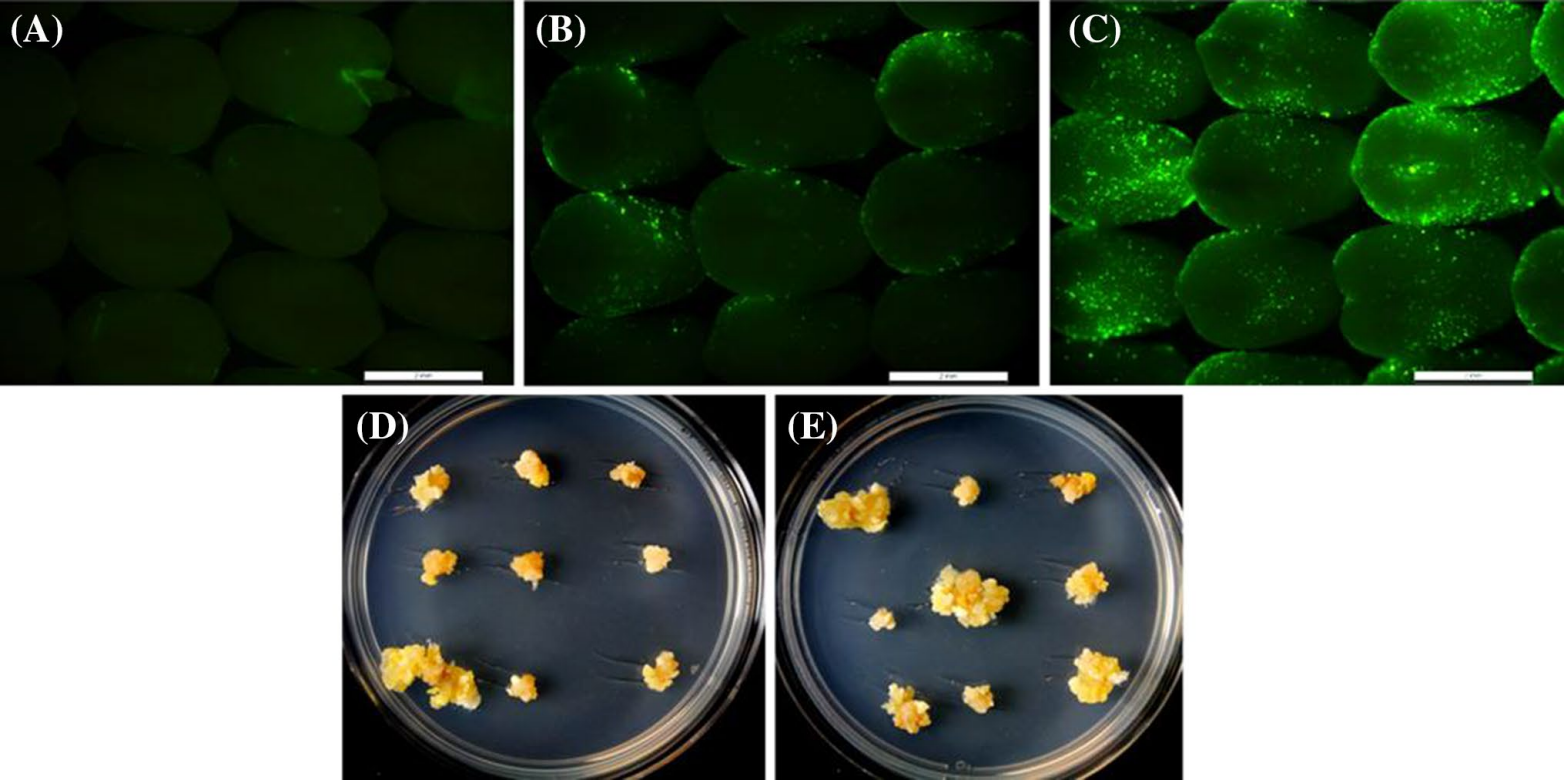
Transient and stable transformation events with the ternary vector containing different accessory plasmids in the maize inbred PH2RT. The top panel displays the transient yellow fluorescent protein expression in immature embryos infected with binary vector pPHP45981 (**a**), ternary vector pSB1/pPHP45981 (**b**) and pPHP70298/pPHP45981 (**c**) 5 days post infection. The bottom panels depict the callus events generated from ternary vectors, pSB1/pPHP45981 plus (**d**), and pPHP70298/pPHP45981 (**e**) 5 weeks post embryo infection. Stronger transient gene expression and higher stable transformation events were detected in embryos transformed with ternary vector containing plasmids pPHP70298/pPHP45981

The utility of the accessory plasmids on maize transformation was analyzed by introducing the accessory plasmids pSB1 or pPHP70298 into *A. tumefaciens* strain LBA4404THY-. Subsequently, a binary plasmid pPHP45981, with repABC (pRi) replicon ORI and spectinomycin bacterial selectable marker harboring a simple T-DNA (7.8 kb) containing a reporter gene (ZMUbi1Pro-Intron:YFP:PINII TERM) and a selectable marker (ZMUbi1Pro-Intron:PMI:PINII TERM) between the borders (Fig. 4), was electroporated into the same *Agrobacterium* cell line (pSB1 or pPHP70298), to generate the ternary constructs, pSB1/pPHP45981 (control) and pPHP70298/pPHP45981. Immature embryo transformation was performed on difficult-to-transform maize inbreds HC69 and PH2RT (Cho et al. 2014; Zhi et al. 2015). To visualize transient T-DNA transformation, the expression of the YFP (ZS-Yellow) reporter gene was observed after 3 and 5 days of co-cultivation. Representative experiments in the inbreds PH2RT and HC69 at 5 days post-infection are shown in Fig. 5 and Supplementary Fig. 3. Stronger YFP expression was seen in embryos infected with pPHP70298/pPHP45981 (Fig. 5c; Supplementary Fig. 3B) as compared to the embryos infected with the control pSB1/pPHP45981 (Fig. 5b; Supplementary Fig. 3A) or binary plasmid pPHP45981 alone (Fig. 5a). The above observation indicated that the accessory plasmid pPHP70298 improved transient T-DNA delivery when compared to pSB1. Importantly, pPHP70298 also increased the recovery of stable callus events compared to the control (Fig. 5d, e; Table 1). Taken together, these results demonstrate pPHP70298 improved both transient and stable T-DNA transformation in maize. We are not sure whether the above improvements resulted from the inclusion of functional *vir* genes (*virC1, virC2, virD1 and virD2*), or the high copy plasmid replicon pVS1, or the additional amendments made in plasmid pPHP70298.

**Table 1:** Transformation and molecular event data in two different maize inbred lines HC69 and PH2RT transformed with *Agrobacterium* strain LBA4404 THY- containing the plasmids pSB1 and pPHP70298 plus a T-DNA binary vector containing the YFP and PMI genes within the T-DNA borders

Inbred	Vectors	Total embryos	Total T0 events	T0 transformation frequency (%) ± standard error	Number of quality events	Quality event frequency (%)	Usable event frequency (%)
HC69	pPHP45981/pSB1	641	90	14.0 ± 1.9	40	44.4	6.5
	pPHP45981/pPHP70298	759	204	26.9 ± 1.2*	65	31.9	9.9
PH2RT	pPHP45981/pSB1	463	207	44.7 ± 0.8	86	41.5	18.6
	pPHP45981/pPHP70298	457	277	60.6 ± 1.4*	53	20.2	12.3

*p < 0.05

^a^Three replicates of each experiment were used to determine the mean transformation frequency with SE values. The percentage of QEs was divided by the total number of events analyzed to calculate the QE frequency. The usable event (UE) frequency is a measure of the number of acceptable transgenic events per 100 embryos that was determined as the product of QE frequency and transformation frequency. Statistical difference was determined by Tukey’s test

Next, we assessed the effect of the accessory plasmids on stable transformation and stable transgene integration. The total number of T0 events was divided by the total number of embryos infected to compute stable transformation frequency. Stable T-DNA integration was confirmed by qPCR (for transgene copy number determination) and multiplex PCR (for detecting vector backbone integration) using genomic DNA extracted from the putative T0 transgenic events as described in Zhi et al. 2015. Events that contained a single copy of the transgenes within the T-DNA and were free of vector backbone integration were identified as quality events (QE). The percentage of QEs was divided by the total number of events analyzed to calculate the QE frequency. The usable event (UE) frequency is a measure of the number of acceptable transgenic events per 100 embryos which was determined as the product of QE frequency and transformation frequency.

Consistent with transient expression, T0 transformation frequency in the inbreds HC69 and PH2RT was significantly higher with pPHP70298/pPHP45981 compared to control pSB1/pPHP45981 (Table 1). In HC69, the raw T0 frequency almost doubled compared to the control (27 vs. 14%), while in PH2RT the increase was significant (61 vs. 45%). However, the QE frequency was found to be lower with pPHP70298/pPHP45981 compared to the control for inbreds HC69 (32 vs. 44%) and PH2RT (20 vs. 42%) (Table 1). This suggests that the improved T0 transformation with accessory plasmid pPHP70298 may have contributed to a higher proportion of non-quality events (non-QE), i.e. events with backbone (BB) integration and/or multi-copy (MC) events. This is consistent with the observation in HC69 for pPHP70298/pPHP45981 that resulted in events with higher frequency of BB integration (22%) and MC (45%) compared to the control (14 and 35%), respectively. A similar pattern was observed for pPHP70298/pPHP45981 compared to the control in the second inbred, PH2RT (BB integration: 20 vs. 12%, MC events: 56 vs. 33%). Next, the UE frequency was calculated for each inbred. The UE frequency was higher than control in HC69 (9.9 vs. 6.5%) and lower than control in PH2RT (12.3 vs.18.6%) (Table 1). Taken together, the use of ternary vectors carrying accessory plasmid pPHP70298 improved the transient and stable transformation frequency in the inbreds HC69 and PH2RT, but resulted in higher percentage of non-QE in the two genotypes. The higher proportion of non-QE with pPHP70298 likely resulted from the increased T-strand delivery (stronger transient gene expression) in maize cells producing complex extrachromosomal T-DNA structures as previously described (Singer et al. 2012) to generate a higher proportion of events with BB integration and multiple copies of the transgenes.

### Plasmids pPHP70298 and pPHP71539 improved maize transformation

Since the observed UE frequency in PH2RT was lower with a ternary vector containing plasmid pPHP70298, we evaluated the accessory plasmid pPHP71539 (Fig. 1c) designed to have additional *vir* genes (VirD, VirE operons) to improve UE frequency on maize transformation. In this experiment, three different accessory plasmids (pSB1, pPHP70298 and pPHP71539) were introduced into *A. tumefaciens* strain LBA4404THY- harboring binary vectors with proprietary trait genes. Three different constructs were selected (A, B and C), each containing a different proprietary trait gene (variable DNA piece), the plant selectable marker phosphomannose-isomerase (PMI), and the herbicide resistance gene phosphinothricin acetyl transferase (moPAT). The size of the T-DNA in these three constructs was 11.5, 13.8 and 16.6 kb. Table 2 summarizes the three-way comparison of the different accessory plasmids and trait constructs A, B and C on transformation and event quality in the inbred PH2RT. Consistent with our previous data, ternary vectors containing accessory plasmids pPHP70298 and pPHP71539 resulted in significantly higher callus transformation frequency and putative T0 transformation frequency compared to pSB1 (Table 2). Plasmid pPHP71539 was the best performing accessory plasmid, averaging 1.6 and 2.3-fold higher callus transformation frequency and putative T0 transformation frequency compared to the plasmids pPHP70298 or pSB1, respectively.

**Table 2:** The effect of different ternary vectors harboring one of the accessory plasmids pSB1, pPHP70298 and pPHP71539 plus binary vector with proprietary trait genes (A, B and C) on callus transformation, transformation frequency and event quality in the inbred PH2RT

Construct	Accessory plasmids	Total embryos	Callus events	Callus transformation frequency (%)	Total T0 events	T0 transformation frequency (%) ± standard error	Total events analyzed	Number of quality events	Quality event frequency (%)	Usable event frequency (%)
A	pSB1	549	120	21.9 ± 3.6	71	12.9 ± 1.6	71	29	40.8	5.3
	pPHP70298	577	213	36.9 ± 5.8*	139	24.1 ± 2.1*	95	25	26.3	6.3
	pPHP71539	546	265	48.5 ± 4.2*	181	33.2 ± 2.8*	95	27	28.4	9.4
B	pSB1	476	113	23.7 ± 4.6	69	14.5 ± 1.9	64	25	39.1	5.7
	pPHP70298	514	166	32.3 ± 3.9*	97	18.9 ± 1.9*	74	24	32.4	6.1
	pPHP71539	514	262	51.0 ± 4.9*	149	29.0 ± 2.2*	97	33	34.0	9.9
C	pSB1	584	135	23.1 ± 3.9	80	13.7 ± 1.1	78	24	30.8	4.2
	pPHP70298	646	199	30.8 ± 4.6*	110	17.0 ± 1.6	92	27	29.3	5.0
	pPHP71539	631	358	56.7 ± 4.6*	196	31.1 ± 2.4*	106	29	27.4	8.5
Total	pSB1	1609	368	22.9 ± 3.9	220	13.7 ± 1.6	213	78	36.6	5.1
	pPHP70298	1737	578	33.3 ± 4.8*	346	19.9 ± 1.9*	261	76	29.1	5.8
	pPHP71539	1691	885	52.3 ± 4.4*	526	31.1 ± 2.5*	298	89	29.9	9.3

Data from three independent transformers was used to determine the callus transformation frequency and T0 transformation frequency. The percentage of QEs was divided by the total number of events analyzed to calculate the QE frequency. The usable event (UE) frequency is a measure of the number of acceptable transgenic events per 100 embryos that was determined as the product of QE frequency and transformation frequency. Statistical difference was determined by Tukey’s test

*p < 0.05

A subset of the putative T0 events was subjected to molecular analysis. The QE frequency with plasmid pSB1 was higher, which is consistent with our earlier observation. Overall the QE frequency was lower with plasmid pPHP71539 (29.9%) when compared to control pSB1 (36.6%) (Table 2). The low QE frequency as compared to control was a result of several factors including higher frequency of events with BB-insertion (19.8 vs. 11.7%), higher proportion of MC events (64 vs. 48%) and higher rates of null events or escapes (8.1 vs. 4.2%) (Supplementary Table 1). Compared to the control, plasmid pPHP70298 also resulted in lower QE frequency (29.1 vs. 36.6%; Table 2), with higher proportion of nulls (6.9%) and MC events (59%, Supplementary Table 1). A possible explanation for the higher frequency of non-QE with pPHP71539, could be due to the presence of extra VirE2 protein produced by the extra copy of the VirE operon in pPHP71539. The additional VirE2 protein would bind and protect extra copies of the T-strands (Christie et al. 1988; Citovsky et al. 1997) which, along with the synthesis of complex extrachromosomal T-DNA structures (Singer et al. 2012) potentially resulted in stable integration of additional copies of the T-strands into the plant chromosome (Supplementary Table 1). The increase in the number of null events could be explained by stable integration and high expression of multiple copies of the selectable marker (PMI). The presence of abundant phosphomannose isomerase protein may have helped protect adjoining non-transformed cells allowing them to escape selection, resulting in the higher number of null events. Interestingly, the observed data on transformation frequency and non-QE frequency is consistent with the previously described work using the hypervirulent strain of *Agrobacterium* AGL0 (Zhi et al. 2015). The hypervirulent strain AGL0 containing helper plasmid pTiBo542∆T-DNA significantly improved transformation frequency but produced a higher frequency of non-QE resulting in overall lower frequency of quality events. A similar effect on non-QE frequency from incorporation of hypervirulence genes from pTiBo542 in pVIR plasmids cannot be ruled out. Although QE frequency is reduced, without this technology it is difficult to get good number of events with proprietary trait gene constructs in the difficult -to -transform maize inbreds. Regardless, the overall UE frequency was almost doubled with plasmid pPHP71539 (9.3%) compared to control pSB1 (5.1%). The introduction of the accessory plasmid pPHP71539 enabled development of a superior ternary vector system for transforming recalcitrant maize inbreds.

An additional benefit to using the pVIR plasmids is the rapid callus growth seen in embryos transformed with plasmids pPHP70298 and pPHP71539, resulting in the events being advanced to maturation stage a week to 10 days earlier than with the control plasmid. We observed higher transient T-DNA delivery and a larger number of cells receptive to T-DNA, resulting in selective advantage for the transformed cells to proliferate on the selection medium compared to the control (pSB1). The putative T0 events were regenerated more rapidly, in total reducing the transformation process by 2–3 weeks. Therefore, the pVIR plasmids not only improved the transformation frequency and UE frequency in both inbreds tested but also shortened the transformation process. These improvements in speed and efficiency on maize transformation using the newly described ternary vector system are highly desirable for high-throughput transformation.

### A rapid maize transformation system for evaluating the effect of plasmid origin of replication on maize transformation

A recently described rapid maize transformation method (Lowe et al. 2018) was used to assess the effect of 13 different ORI-by-ORI combinations (as listed in Table 3) on maize transformation. The accessory plasmid pPHP71539 was critical for developing rapid maize transformation technology with high transformation frequency in multiple difficult to transform maize inbreds (Lowe et al. 2018). Briefly, the rapid method of transformation relies on expression of morphogenic genes *Baby boom* (*Bbm*) and *Wuschel2* (*Wus2*) (Lowe et al. 2016), allowing quicker recovery of transgenic maize plants at high frequency from immature embryo transformation. The method significantly reduces the number of steps involved in transformation, directly forming somatic embryos with no intermediate callus phase required. Transformed plants can be germinated rapidly, requiring a month from infection to sending plants to the greenhouse. This method results in much higher transformation frequencies compared to conventional *Argobacterium*-mediated transformation and is ideal for rapid gene-function analysis and developing swift crop transformation technologies.

**Table 3:** The effect of ternary vectors on transformation frequency containing different ORI-by-ORI combinations of the accessory plasmid and the T-DNA containing binary vector in the inbred HC69

Group	Construct number	ORI combination	Binary/pVIR combinations	Total embryos	Total T0 plants	Transformation frequency (%) ± standard error
1	1	pVS1/pRi	RV007067/RV002110	160	104	65 ± 5.2^bc^
	2	pVS1/pSa	RV007067/RV005072	164	111	68 ± 5.2^b^
	3	pVS1/RK2	RV007067/RV005072	165	120	73 ± 5.2^b^
	Control	pRi/pVS1	RV001750/PHP71539	161	153	95 ± 5.2^a^
2	4	pSa/pRi	RV007069/RV002110	170	134	79 ± 6.1^b^
	5	pSa/pVS1	RV007069/RV004296	170	146	86 ± 6.1^a^
	6	pSa/RK2	RV007069/RV005392	170	123	72 ± 6.1^b^
	Control	pRi/pVS1	RV001750/PHP71539	170	165	97 ± 6.1^a^
3	7	RK2/pRI	RV007070/RV002110	176	116	66 ± 8.7^b^
	8	RK2/pVS1	RV007070/RV004296	166	58	35 ± 8.7^c^
	9	RK2/pSa	RV007070/RV005072	183	164	90 ± 8.7^a^
	Control	pRi/pVS1	RV001750/PHP71539	175	161	92 ± 8.7^a^
4	10	pRi/pVS1	RV001750/RV004296	183	154	84 ± 7.6^ab^
	11	pRi/pSa	RV001750/RV005072	160	116	73 ± 7.6^b^
	12	pRi/RK2	RV001750/RV005392	176	173	98 ± 7.6^a^
	Control	pRi/pVS1	RV001750/PHP71539	170	175	103 ± 7.6^a^

A total of 13 different ORI-by-ORI combinations were tested using a rapid maize transformation protocol

The effect of 13 ORI-by-ORI combinations on transformation was performed in four groups including the control. The transformation data was analyzed by mixed model using SAS (SAS Institute) and analysis conducted by group. Sample means with same letter were not significantly different at *p* < 0.05

**Fig. 6:**
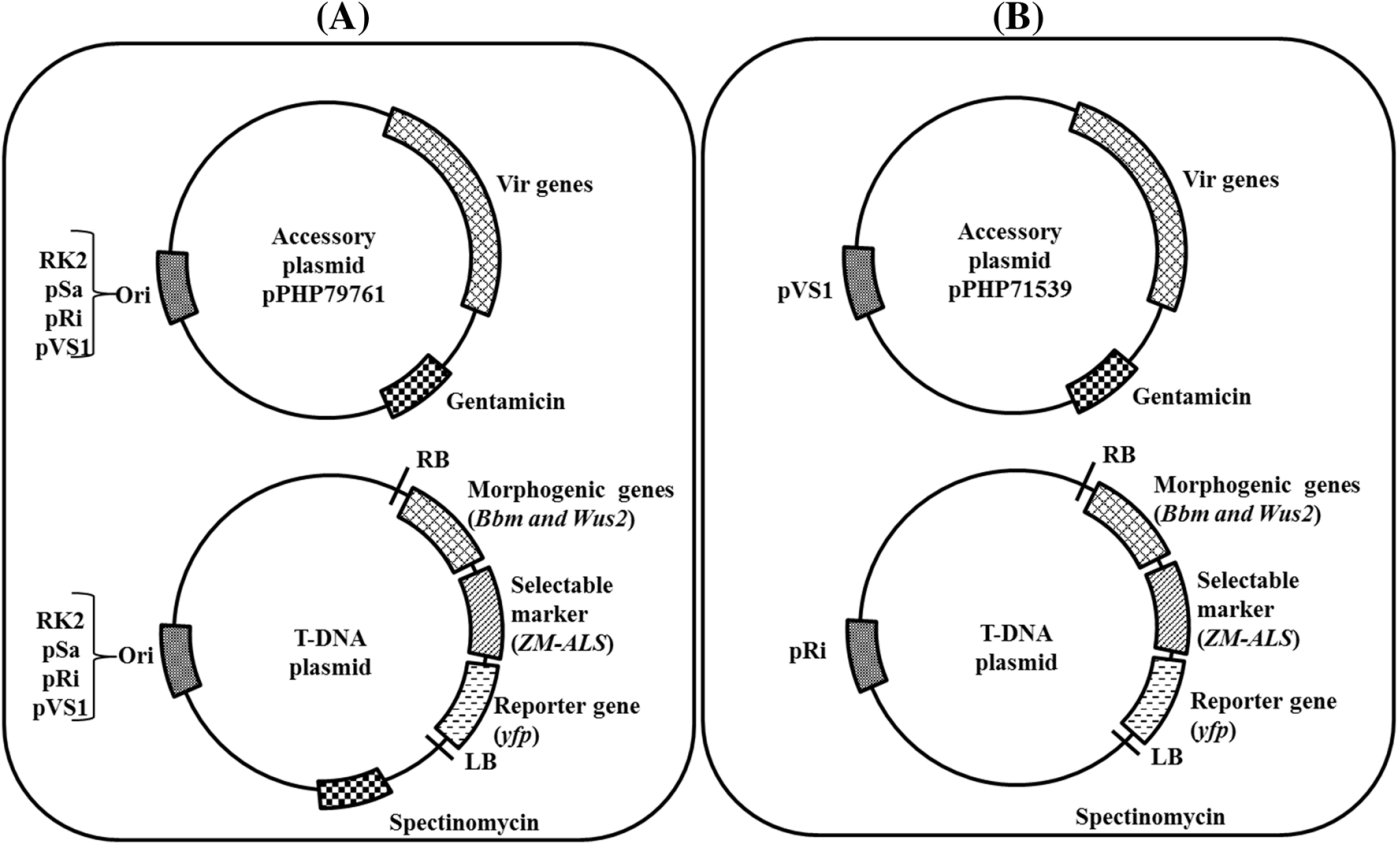
Schematic representation of the different ORI-by-ORI designs tested in the rapid maize transformation system. **a** The ternary vector design used for evaluating 12 different ORI-by-ORI designs containing the binary plasmid and accessory plasmid based on pPHP79761 differing in their replicons. **b** The ternary vector design with pRi (binary plasmid)/pVS1 (accessory plasmid pPHP71539) replicon used as the control in the experiments

Using this rapid maize transformation method, four different bacterial ORI were tested (pVS1, pRi, pSa and RK2; Table 3). These ORI maintain different plasmid copy numbers, ranging from 2 to 3 copies (pRi, pSa), 10–12 copies (RK2) and ~ 20 copies (pVS1) per bacterial cell (Oltmanns et al. 2010; Zhi et al. 2015). Four binary vectors with different bacterial ORI were built, RV001750 (pRi), RV007067 (pVS1), RV007069 (pSa) and RV007070 (RK2), each harboring a 13.8 kb T-DNA containing a WUS2 cassette, a BBM cassette, a selectable marker gene (SB-ALS Pro: ZM-ALS: SB-PEPCI TERM) and a reporter gene (LTP2 Pro: YFP: PinII TERM). HRA is a modified acetolactate synthase gene (ALS) conferring a high level of resistance to imidazolinone and sulfonylurea herbicides (Green et al. 2009). Similarly, four different pVIR accessory plasmids based on pPHP79761 (Fig. 1d) with different ORI [namely RV002110 (pRi), RV004296 (pVS1), RV005072 (pSa) and RV005392 (RK2)] were built and mobilized into *Agrobacterium* strains harboring the binary vectors as detailed in Table 3. A schematic drawing of the different ORI-by-ORI (binary vector-by- accessory plasmid with different ORI) plasmid combination is presented in Fig. 6. The 12 ORI-by-ORI (Fig. 6a) combinations were tested against the control, pPHP71539/RV001750 (Fig. 6b), in the inbred HC69 using ethametsulfuron (0.1 mg/L) herbicide selection to determine the effect of the two ORIs and their interaction on rapid maize transformation. The results from this study are summarized in Table 3. All 13 ORI-by-ORI combinations supported rapid maize transformation, producing T0 plants within a month following embryo infection. Transformation frequencies ranged from 65 to 103%, with the exception of the RK2/pVS1 combination that produced 35% (Table 3). Seven ORI-by-ORI combinations (pVS1/pRi, pVS1/pSa, pVS1/RK2, pSa/pRi, pSa/RK2, RK2/pRi and pRi/pSa) resulted in significantly lower transformation frequency compared to the control (Table 3). We also identified four ORI-by-ORI combinations pSa/pVS1, RK2/pSa, pRi/RK2 and pRi/pVS1 (control, pPHP71539/RV001750) that resulted in very high raw transformation frequencies (86%-103%, Table 3). When compared to conventional transformation in HC69 with pSB1 (Table 1), this represents a six-to-seven-fold improvement. Based on these data, we concluded that pVS1 ORI on the pVIR plasmid and pRi ORI on the binary plasmid performed reasonably well with different ORI combination (except for the RK2 ORI-based binary vector). To the contrary, use of pSa and RK2 in either pVIR/binary combination was identified to be a poorly performing combination. In a previous study we showed direct correlation between binary plasmid copy number on transformation frequency and UE (Zhi et al. 2015). However, in the rapid transformation method an opposite trend was observed with pRi ORI (low copy) on the binary plasmid, resulting in higher transformation frequency. Improved transformation frequency was generally observed when the pVIR plasmid contained the high copy ORI (pVS1), with one exception (pVS1/RK2), suggesting that the choice of ORI is important for maize transformation. The work presented here and the recent work by Lowe et al. (2018) potentially opens the possibility for developing a genotype-independent transformation in maize by modulating the expression of the morphogenic genes along with the use of accessory plasmid pPHP71539. We recently reported the application of the accessory plasmid in a ternary vector for developing a highly efficient transformation system in sorghum (Che et al. 3) and are further testing it for improving transformation of multiple cereal crops including wheat and rice.

## Experimental procedures

### Construction of the pVIR plasmids

The pVIR plasmids, pPHP70298 (Fig. 1b), pPHP71539 (Fig. 1c) and pPHP79761 (Fig. 1d) were built in very similar manner. The construction of pPHP79761 is described here as an example of the methods used in the construction of pPHP70298 and pPHP71539. The virulence genes were identified and selected from the Ti plasmid, pTiBo542 (GenBank Accession No. NC_010929.1; Fig. 2). PCR primers were designed to amplify the *virA, virJ*, and *virB1*-*virE3* coding and regulatory regions from a genomic DNA prep of *A. tumefaciens* strain AGL1. The 24 kb *virB1-E3* sequence was amplified in 5 pieces to facilitate PCR. Fragments were designed with 40 bp overlapping ends to facilitate seamless cloning methods. Unique restriction enzyme sites were included between functional elements to facilitate their exchange with other functionally equivalent elements as shown in Fig. 1d. A gentamicin acetyltransferase gene (GenBank Accession No. DQ530421) conferring gentamicin resistance was synthesized with flanking restriction sites introduced upstream of the *vir* genes. The high copy ColE1 ORI of pBR322 (GenBank Accession No. J01749) was included for stable replication in *E. coli* and the pVS1 ORI was included for stable maintenance in *Agrobacterium*.

The pVIR plasmids were mobilized into an auxotrophic *Agrobacterium* strain LBA4404THY- by electroporation and the recombinant colonies were selected on minimal medium supplemented with 12.5 mg/L tetracycline (pSB1) or 25 mg/L gentamicin (pPHP70298, pPHP71539 and pPHP79761). For conventional maize transformation, only plasmids pSB1, pPHP70298 and pPHP71539 were tested with a research vector and three different trait gene vectors. In the research vector, the maize ubiquitin (Ubi-1) promoter with its intron1 was used to drive two different gene cassettes, a yellow fluorescent reporter gene (YFP), and phosphomannose isomerase (PMI) (Negrotto et al. 2000), fused to the PinII terminator (Fig. 4). These cassettes were generated as separate entry clones and introduced into a Gateway™ binary vector with *att*R4 and *att*R3 sites, pRi ORI and spectinomycin as the bacterial selectable marker on the vector backbone. Additionally, three more constructs (A, B and C), each containing different proprietary trait gene (variable DNA piece), a maize ubiquitin (Ubi-1) promoter with its intron1 driving the phosphomannose-isomerase (PMI) fused to the PinII terminator and a rice action (Os-Act) promoter with its intron driving a maize codon-optimized phosphinothricin acetyltransferase (moPAT) (Jayne et al. 2000) fused to the CaMV35S terminator within the T-DNA borders were generated. The T-DNA containing binary vectors were mobilized into strain LBA4404 THY- harboring the plasmids (pSB1, pPHP70298 and pPHP71539) by electroporation and the resultant recombinant colonies were selected on appropriate antibiotic (rifampicin, 10 mg/L; spectinomycin,100 mg/L; tetracycline 12.5 mg/L or gentamicin 25 mg/L) supplemented media. All plasmids in *Agrobacterium* used plant transformation were sequenced verified by next gen sequencing (Illumina) prior to transformation. Some materials reported in this paper contained the selectable markers moPAT and PMI owned by third parties. Pioneer Hi-Bred International, Inc. (Pioneer) will provide materials to academic investigators for non-commercial research under an applicable material transfer agreement subject to proof of permission from any third-party owners of all or parts of the material and to governmental regulation considerations. Obtaining permission from third parties will be the responsibility of the requestor.

To generate various pVIR plasmids with different ORI, we replaced the pVS1 ORI in pPHP79761 with ORI RK2, pSa and pRi using seamless cloning strategy. Likewise, the binary vector with the pRi ORI harboring a T-DNA (13.8 kb, unpublished) containing a WUS2 cassette, a BBM cassette, a sorghum ALS promoter driving modified *Zea mays* acetolactate synthase gene (ZM-ALS) fused to a sorghum PEPCI terminator and a lipid transfer protein promoter driving a yellow fluorescent protein gene (YFP) fused to the PinII terminator was replaced with RK2 ORI, pSa ORI and pRi ORI using a seamless cloning strategy. The pVIR plasmids and binary vectors transformed into *Agrobacterium* strain LBA4404THY- were selected on appropriate antibiotic selection and sequence verified by next gen sequencing (Illumina).

### *Agrobacterium tumefaciens* culture conditions

*Agrobacterium tumefaciens* strains were grown on solidified or liquid AB Sucrose or yeast peptone medium (YP) or Luria Broth (LB) medium supplemented with appropriate antibiotics (rifampicin, 10 mg/L; spectinomycin,100 mg/L; tetracycline 12.5 mg/L or gentamicin 25 mg/L) and thymidine (50 mg/L) at 28 °C. For plant transformation *Agrobacterium* strains were streaked from glycerol stocks on AB sucrose media supplemented with appropriate antibiotics; 1–3 colonies were picked 2–3 days post plating and streaked on freshly prepared YP media as previously described.

### Analysis of plasmid stability

Two independent colonies of *E. coli* and *Agrobacterium* (LBA4404THY-) harboring plasmids pPHP70298 and pPHP71539 cultured on LB Agar and antibiotic selection, were used to inoculate 10 ml of LB with appropriate antibiotic, and incubated at 37 °C or 28 °C with shaking. Of this overnight culture 300 µl was used to re-inoculate 3 ml of LB in the presence/absence of antibiotic selection; at the same time a serial dilution of the bacteria grown under non-selective media was plated onto selective and nonselective media. The total viable count and antibiotic resistant bacterial count was used to calculate the mean colony-forming units per ml of culture, using the data from the dilution series. The inheritance of plasmid was monitored for four passages (overnight incubations). For plasmid integrity, the plasmid DNA from *Agrobacterium* cultures grown in the presence of antibiotic for four passages were retransformed into *E. coli*, followed by two passages and the pooled DNA subjected to restriction enzyme analysis. The banding pattern was used to infer the plasmid integrity after each growth passage. The same plasmid DNA was subjected to next gen sequencing for comparing the expected and observed plasmid DNA sequences.

### Maize transformation and molecular analysis

Immature embryos derived from commercial elite inbred lines HC69 and PH2RT, were used for *Agrobacterium*-mediated transformation. Maize immature embryo transformation was performed using split ear transformation as described in Cho et al. (2014) using PMI with mannose selection. The T0 transformation frequency was determined as the number of events (plantlet with roots) produced to the total number of embryos infected. Only a single healthy looking event per embryo was used for determining the transformation frequency and for molecular analysis. For the ORI-by-ORI analysis in rapid maize transformation method transformation, the immature embryos were harvested from maize inbred (HC69), infected with *Agrobacterium* strain containing the ORI-by-ORI combinations using the transformation protocol described by Lowe et al. (2018) was used. Four independent transformation experiments were conducted for each ORI-by-ORI combination, and transformation frequencies were determined by analyzing all the events regenerated per construct.

Genomic DNA extracted from leaf slices derived from putative T0 events and wild-type non-transgenic PHR03 was used for determining the molecular event quality by quantitative PCR (qPCR) as previously described (Wu et al. 2014). We determined the copy number of T-DNA genes (PMI, YFP, moPAT and trait gene within the T-DNA borders) using qPCR and used a multiplex backbone PCR assay for detecting the presence/absence of the binary vector backbone (Wu et al. 2014). Events identified with a single copy (SC) of all the transgenes without vector backbone integration were defined as a quality event (QE). The usable event (UE) frequency was calculated as transformation frequency times QE frequency.

### Statistical analysis

Data for the construct pPHP45981was collected following split ear transformation, with a minimum of 400 embryos, involving at least two independent researchers and three independent experiments. The T0 transformation frequency (stable events/embryos infected) was considered the response variable, with individual researchers as a fixed effect. The data on transformation frequency was collected for each inbred, mean and standard error values were determined. For trait gene (A, B and C) constructs involving three-way testing of the accessory plasmids, split ear transformation was performed on a minimum of four ears per construct involving three independent transformers. A minimum of 500 embryos/production construct was infected. For each individual construct callus transformation frequency and T0 transformation frequency was considered variable while the effect of different constructs/accessory plasmids on transformation rates was not compared. The mean callus and transformation frequency with standard error values was calculated and statistical significance determined by Tukey’s test. In the ORI-by-ORI testing, a minimum of 30 embryos were infected by two independent transformers, replicated twice to determine the effect of the construct on putative T0 transformation frequency. The transformation data was analyzed by mixed model using SAS (SAS Institute) and analysis conducted by group. Both transformer and vectors were included as fixed effect in the model. LS-mean for each vector was generated from the model. DUNNETT was used for multiple comparison adjustments on the differences of LS-Means. The significant level was controlled as *p* < 0.05.

## Electronic supplementary material

Below is the link to the electronic supplementary material.

Supplementary material 1 (DOCX 717 KB)

## Abbreviations

QE: Quality events
UE: Usable event
ORI: Origin of replication
Bbm: Babyboom
Wus2: Wuschel
Vir: Virulence
PMI: Phosphomannose isomerase
moPAT: Phosphinothricin acetyl transferase
HRA: Modified acetolactate synthase
