# Supplementary material for: An improved ternary vector system for Agrobacterium-mediated rapid maize transformation
Source: Plant Mol Biol. 2018 Apr 23;97(1):187–200. doi: 10.1007/s11103-018-0732-y (PMC5945794; doi:10.1007/s11103-018-0732-y)
Supplement: Supplementary file 1 — Supplementary material 1 (DOCX 717 KB) [file 11103_2018_732_MOESM1_ESM.docx]

**Supplementary Figure 1.** Schematic presentation of the ternary vector designs used for maize transformation. A) Ternary vector containing accessory plasmid pSB1 and a T-DNA binary vector and B) ternary vector containing plasmid pPHP70298 and a T-DNA binary vector.


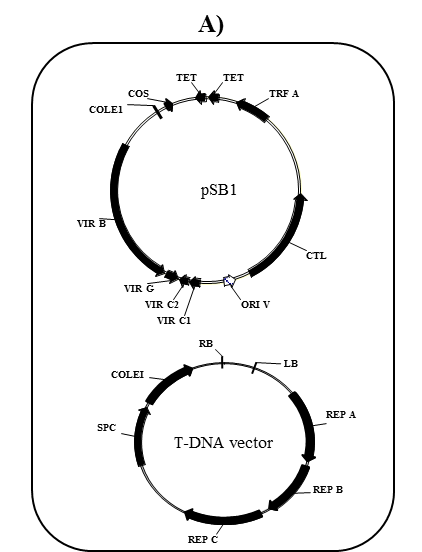

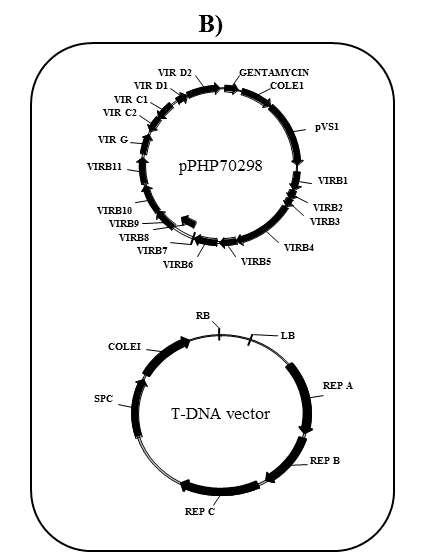


**Supplementary Figure 2.** Next gen sequencing of plasmid DNAs pPHP70298 and pPHP71539 in *Agrobacterium*. The pooled plasmid DNA extracted from *Agrobacterium* strain LBA4404Thy- containing pPHP70298 or pPHP71539 after four passages in antibiotic supplemented media and retransformed in *E.coli* was subjected to next gen sequencing (Illumina). The observed sequence matched with the expected sequence and no SNPs were detected, confirming plasmid integrity at base pair level.

A)


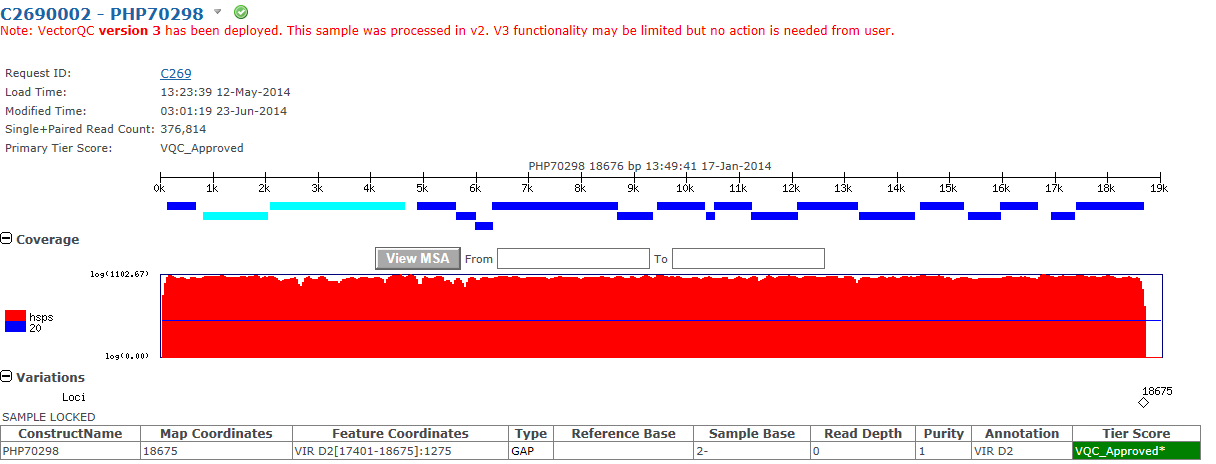


B)


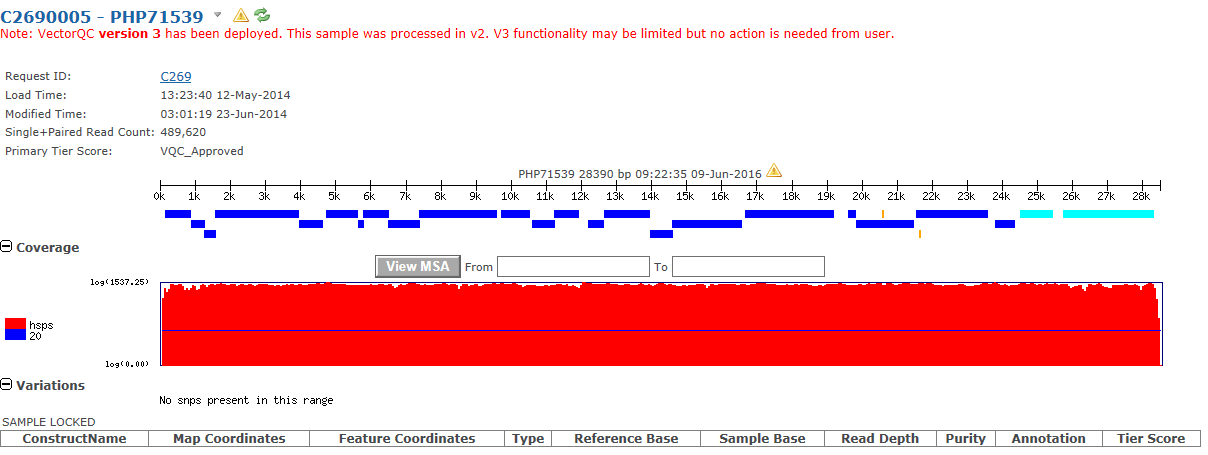


**Supplementary Figure 3.** Transient gene expression in the inbred HC69. The transient yellow fluroscent protein expression in immature embryos infected with ternary vector pSB1/pPHP45981(A) and pPHP70298/pPHP45981(B) at three days post infection.


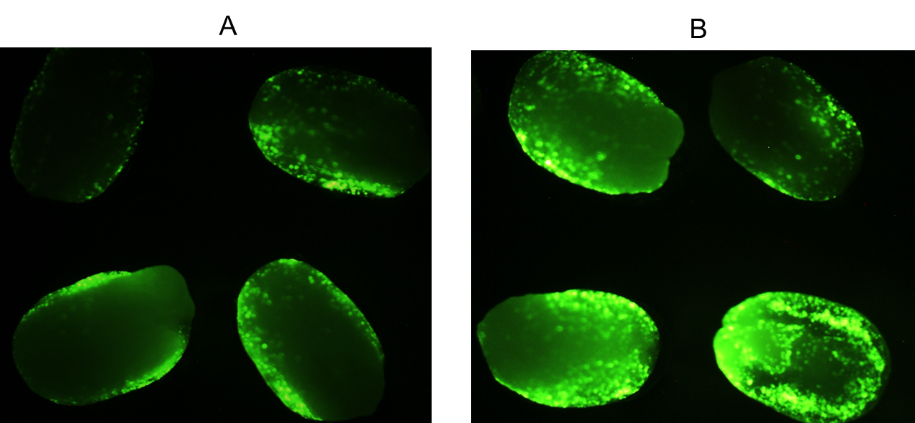


Supplementary Table 1. The breakdown of non-quality events by the proportion of multi-copy, backbone integration

and null events in maize inbred PH2RT transformed with ternary vectors harboring binary vectors containing

properitary trait genes and different accessory plasmids.

| Construct | Vectors | Total events analyzed | Total Multi copy events | Multi copy event frequency (%) | Number of null events | Null event frequency (%) | Total backbone positive events | Backbone positive event frequency (%) |
| --- | --- | --- | --- | --- | --- | --- | --- | --- |
| A | pSB1 | 71 | 33 | 46.5 | 2 | 2.8 | 7 | 9.9 |
|  | pPHP70298 | 95 | 59 | 62.1 | 6 | 6.3 | 14 | 14.7 |
|  | pPHP71539 | 95 | 58 | 61.1 | 10 | 10.5 | 16 | 16.8 |
| B | pSB1 | 64 | 31 | 48.4 | 5 | 7.8 | 7 | 10.9 |
|  | pPHP70298 | 74 | 42 | 56.8 | 7 | 9.5 | 4 | 5.4 |
|  | pPHP71539 | 97 | 60 | 61.9 | 7 | 7.2 | 13 | 13.4 |
| C | pSB1 | 78 | 39 | 50.0 | 2 | 2.6 | 11 | 14.1 |
|  | pPHP70298 | 92 | 53 | 57.6 | 5 | 5.4 | 14 | 15.2 |
|  | pPHP71539 | 106 | 71 | 67.0 | 7 | 6.6 | 30 | 28.3 |
| Average | pSB1 | 213 | 103 | 48.4 | 9 | 4.2 | 25 | 11.7 |
|  | pPHP70298 | 261 | 154 | 59.0 | 18 | 6.9 | 32 | 12.3 |
|  | pPHP71539 | 298 | 189 | 63.4 | 24 | 8.1 | 59 | 19.8 |
